# Particles and corrected particles of LDL and non-HDL are stronger predicters of coronary lesion in postmenopausal women

**DOI:** 10.1186/s12872-021-02189-x

**Published:** 2021-08-02

**Authors:** Chuang Li, Jingxun Chen, Siyue Wei, Mei Zhang, Yushun Chu, Fanpeng Meng, Jianyu Wang, Jie Tang, Jian Luo, Yu Yang, Xiulong Niu, Wei Cai

**Affiliations:** 1 Department of Thoracic and Cardiovascular Surgery, Special Medical Center of Chinese People’s Armed Police Force, 220 Chenglin Road, Tianjin, 300162 China; 2 School of Engineering, Chinese University of Hong Kong, Hong Kong, China; 3 W.P. Carry School of Business, Arizona State University, Tempe, Arizona USA; 4 Tianjin Beichen Hospital, Beichen Tianjin, China

**Keywords:** Coronary heart disease, Lipid test, Nuclear magnetic resonance spectroscopy, Postmenopausal females, Low-density lipoprotein cholesterol

## Abstract

**Background:**

The optimum lipid indexes, predicting the coronary lesion in postmenopausal women are not clear.

**Objective:**

To evaluate the optimum lipid predicter for coronary lesion in routine and advanced lipid tests.

**Method:**

300 postmenopausal women were enrolled and assigned into coronary heart disease (CHD) Group (242), and non-CHD Group (58). Routine and advanced lipid indexes were measured with standard laboratory test and nuclear magnetic resonance (NMR) spectroscopy. The correlation and predictivities for CHD of routine and advanced lipid indexes were performed with Logistic regression, Spearman correlation analysis and receiver operating characteristic (ROC).

**Results:**

Age (hazard ratio (HR) 2.58, 95% confidence interval (CI) 1.08–5.86, *P* = 0.03), apolipoprotein B (ApoB) (HR 1.35, 95% CI 1.15–1.59, *P* < 0.001), corrected particles of low-density lipoprotein (LDL-p-corr) (HR 1.05, 95% CI 1.03–1.06, *P* < 0.001) and corrected particles of non-high-density lipoprotein (non-HDL-p-corr) (HR 1.02, 95% CI 1.01–1.03, *P* < 0.001) were the risk factors of CHD. LDL cholesterol (LDL-C), LDL-p, LDL-p-corr, HDL cholesterol (HDL-C), non-HDL cholesterol (non-HDL-C), non-HDL-p and non-HDL-p-corr were in linear correlation with Gensini score. Advanced lipid indexes LDL-p (area under curve (AUC) = 0.750, *P* = 0.02), LDL-p-corr (AUC = 0.759, *P* = 0.02), non-HDL-p (AUC = 0.693, *P* = 0.03) and non-HDL-p-corr (AUC = 0.699, *P* = 0.03) were more predictive for CHD than the routine ones (LDL-C and non-HDL-C).

**Conclusion:**

In postmenopausal women, age, ApoB, LDL-p-corr and non-HDL-p-corr were risk factors of CHD. Compared with traditional lipid items, LDL-p, LDL-p-corr, non-HDL-p and non-HDL-p-corr may be better lipid indexes for CHD in postmenopausal women.

## Background

Cardiovascular disease (CVD) is the leading cause of death in women [1]. Dyslipidemia, especially high-level low-density lipoprotein cholesterol (LDL-C) plays an important role in the atherosclerotic cardiovascular disease (ASCVD) [2, 3]. Postmenopausal women are a special group of females, featured with increased LDL-C level and CVD risk [4, 5]. Hormonal changes, estradiol (E2) mainly, are the partly cause of increased CVD risk for postmenopausal women. E2 is produced primarily in the ovaries with the substrate of LDL-C, and would decline up to 10 pg/mL in postmenopausal from 100 to 250 pg/mL in the fertile life [6]. Thus, postmenopausal women are at higher risk of developing ASCVD with increased LDL-C. As women live longer than men and average life span of women increasing, it was reported that one-third of women will live in postmenopausal state and the rate will be much higher by 2025 in developed countries [7]. As the recently published paper showed a growing prevalence of coronary heart disease (CHD) in women, and women have higher short- to medium-term mortality after myocardial infarction (MI) compared with men [8]. More attention should be paid to the prevention of ASCVD in women, especially in postmenopausal women.

The 2019 ESC/EAS Guidelines for the management of dyslipidemias [9] recommended total cholesterol (TC), LDL-C, high-density lipoprotein cholesterol (HDL-C) and triglycerides (TG) as the routine lipid panel for all people and non-HDL-C for patients with high TG level, very low level of LDL-C, diabetes mellitus (DM) or obesity (I, C). But the routine lipid panel, lack of lipoprotein a (Lp(a)) and particles of lipoprotein, is not efficiency in ASCVD prevention, especially for postmenopausal women. It had been reported that Lp(a), which couldn’t distinguish from LDL-C in routine lipid panel, was an independent risk factor for the presence and severity of new-onset coronary atherosclerotic disease (CAD) in postmenopausal women [10]. A series studies on the characteristics of HDL subgroups in postmenopausal women revealed that smaller HDL size and lower total and medium HDL particles (HDL-p) were associated with incident CVD, whereas lower large HDL-p was associated with extent of coronary calcification [11–13]. The particles of LDL (LDL-p) also appear to be a very strong predictor for CAD in women. In the Cardiovascular Health Study [14], LDL-p and smaller LDL-p size were associated with the incidence of CAD, especially in elderly women. Mackey et al.[13] also reported that LDL-p, small, dense LDL, and large very-low-density lipoprotein (VLDL) were positively associated with coronary artery calcification in healthy postmenopausal women. The last four studies were all based on nuclear magnetic resonance (NMR) spectroscopy, which is an advanced lipid measurement, can provide up to 112 lipid subclasses, 15 metabolites and 9 amino acids in one test. We performed this study in a group of postmenopausal women to find the optimum lipid indexes for predicting coronary atherosclerotic burden with the comparison of routine and advanced lipid items.

## Method

### Study populations

Postmenopausal inpatients, matching the inclusion and exclusion criteria, were enrolled in this study. Inclusion criteria: 1. Female, age > 46 years, natural menopause over 1 year; 2. Conducted CAG between June 2019 and June 2020; 3. All necessary data for this study were available; 4. Clear awareness, able to sign informed consent and willing to take blood measurements. Exclusion criteria: 1. Previous percutaneous coronary intervention (PCI) or coronary artery bypass graft (CABG); 2. Ovariectomy on either side; 3. Under taking lipid lowering medication; 4. Under taking estrogen replacement medication; 5. Cirrhosis or decompensated liver function (Child–Pugh Score > 6); 6. Abnormal renal function (estimated glomerular filtration rate < 60 mL/min/1.73 m^2^); 7. Unstable hemodynamics or left ventricular ejection fraction (LVEF) less than 30%; 8. Rheumatoid or systemic diseases such as sepsis; 9. Severe progressive diseases such as tumors. This study is approved by the ethics committee and conducted with signed consent of all participants in accordance with the Helsinki Declaration.

### Study design

**Fig. 1:**
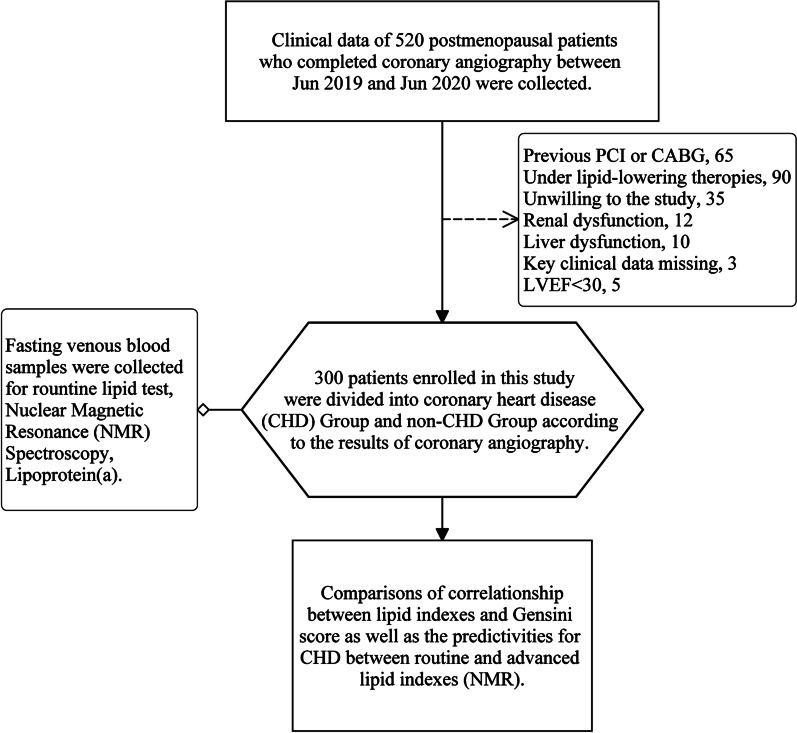
Flow chart

This was a single-center cross-sectional clinical study. Patients with any coronary stenosis ≥ 50% were assigned into CHD Group, with all coronary stenosis < 50% were assigned into non-CHD Group, according to CAG [15] (Fig. 1) . The Gensini scores were calculated based on CAG. The clinical characters were collected from medical record system and blood samples were collected for routine lipid test, NMR spectroscopy and Lp(a) once included in this study. Clinical characters and lipid index were compared between the two groups.

### CAG and Gensini score

The CAGs were performed for patients who had typical or untypical unstable angina pectoris along with myocardial ischemic changes in electrocardiogram, for example, single or multiple leads/territories ST-segment depression/elevation ≥ 1 mm, increased hyperacute T wave amplitude with prominent symmetrical T waves, pathologic Q waves, intraventricular bundle branch blocks, atrioventricular conduction delays, loss of precordial R wave amplitude, etc. Coronary plaque burden was evaluated by Gensini Score [16], which could qualify the severity of the coronary lesions by 3 main parameters: severity score, region multiplying factor and collateral adjustment factor.

### Routine and advanced lipid parameters measurement and calculation

Fasting venues blood samples were collected the day before CAG, and stored at − 70 °C after centrifugation. TC, TG, HDL-C, apolipoprotein A1 (ApoA1) and apolipoprotein B (ApoB) were tested in clinical laboratories with standard measurements. LDL-C was calculated with Friedewald formula. The particles of Lp(a) (Lp(a)-p) were measured in nmol/L by particle-enhanced turbidimetric immunoassay with Tina-quant Lipoprotein (a) Gen.2 (Latex) (LPA2) Roche^®^ on Cobas system. The mass of Lp(a) was calculated with Lp(a)-p * 0.4167[17]. The total particles of cholesterol (TC-p), LDL-p and HDL-p, as well as the average size of LDL were measured with NMR (AvanceIII IVDr, ProteinT^®^, Bruker^®^). Non-HDL-C = TC – HDL-C. The corrected LDL-C (LDL-C-corr) = LDL-C – 0.3 * Lp(a) [18, 19]. The corrected non-HDL-C (non-HDL-C-corr) = non-HDL-C – 0.3 * Lp(a). The particles of non-HDL (non-HDL-p) = TC-p – HDL-C-p. The corrected particles of LDL (LDL-p-corr) = LDL-p – Lp(a)-p. And the corrected particles of non-HDL (non-HDL-p-corr) = non-HDL-p – Lp(a)-p.

### Statistical analysis

Categorical variables were presented as n (%), and the differences between groups were assessed with chi-square test. The continuous variables were tested with Kolmogorov–Smirnov for the normality of distributions and presented as mean ± standard deviations when normally distributed, medians (25^th^, 75^th^ percentile) when non-normally distributed. Student’s *t*-test was used to assess the differences of continuous variables between groups, among which, the non-normally distributed ones were converted into natural logarithm before assessment. Multivariate Logistic regression analysis was performed for CHD risk factors detection by using forward stepwise selection process. Associations between variables and Gensini were assessed using Spearman correlation analysis. Receiver operating characteristic (ROC) for CHD were performed with routine and advanced lipid indexes, and the area under curve (AUC) of advanced lipid items were compared with the routine ones. All statistical analysis was performed with Stata version 15. Data were considered statistically significant when *P* value was less than 0.05.

## Result

### Clinical demography characteristics, routine and advanced lipid parameters

300 patients were enrolled in this study, of which 58 patients were assigned into non-CHD Group. (Table 1) In the clinical demography comparisons, age and TIMI flow 0– were the variables with difference between two groups. Routine lipid indexes, TG, HDL-C and advanced lipid indexes, ApoB, TC-p, Lp(a)-p, Lp(a), LDL-size, HDL-p, non-HDL-C-corr were distributed on the skew. ApoB, TC-p, LDL-p, LDL-p-corr, non-HDL-C, non-HDL-C-corr, non-HDL-p and non-HDL-p-corr in CHD group were significantly higher than in non-CHD group.

**Table 1:** Clinical demography characteristics, routine and advanced lipids

	Non-CHD group (n = 58)	CHD group (n = 242)	*T*/*χ*^*2*^	*P*
Age, y	58.86 ± 8.03	65.76 ± 8.46	5.628	< 0.001
Smoking, No	7 (12.07%)	18 (7.44%)	1.708	0.252
Overweight ^a^, No	19 (32.76%)	65 (26.86%)	1.327	0.369
Diabetes, No	19 (32.76%)	104 (42.98%)	2.022	0.155
Hypertension, No	34 (58.62%)	143 (59.09%)	0.981	0.948
Family history ^b^, No	11 (18.97%)	37 (15.29%)	1.297	0.493
TIMI Flow 0-I ^c^, No	0 (0.00%)	32 (13.22%)	8.585	0.003
TG (mg/dL)*	133.92 (106.85, 200.46)	148.82 (100.94, 209.08)	0.394	0.694
ApoA1 (mg/dL)	134.31 ± 15.94	130.41 ± 15.74	1.693	0.092
ApoB (mg/dL)*	77.91 (58.51, 89.86)	82.10 (68.75, 98.49)	2.839	0.005
TC-p (nmol/L)*	1416.58 (1063.81, 1633.81)	1492.63 (1250.06, 1790.84)	2.839	0.005
TC (mg/dL)	168.16 ± 38.43	177.74 ± 38.68	1.696	0.091
Lp(a)-p (nmol/L)*	42.80 (16.35, 93.88)	36.40 (13.40, 80.00)	0.801	0.424
Lp(a) (mg/dL)*	17.83 (6.81, 39.12)	14.96 (5.42, 33.12)	0.801	0.424
LDL-p (nmol/L)	1042.17 ± 360.58	1168.91 ± 366.35	2.374	0.018
LDL-C (mg/dL)	81.15 ± 32.77	87.30 ± 30.60	1.354	0.177
LDL-size (nm)*	20.44 (20.23, 20.68)	20.37 (20.17, 20.61)	1.634	0.103
LDL-p-corr (nmol/L)	977.50 ± 357.33	1108.44 ± 366.46	2.456	0.015
LDL-C-corr (mg/dL)	73.10 ± 32.30	79.76 ± 30.41	1.481	0.140
HDL-p (nmol/L)*	73.35 (45.78, 121.62)	78.87 (51.55, 114.60)	0.747	0.455
HDL-C (mg/dL)*	45.81 (38.86, 55.28)	45.23 (40.46, 50.51)	0.877	0.381
Non-HDL-p (nmol/L)	1300.29 ± 381.68	1463.23 ± 424.55	2.675	0.008
Non-HDL-p-corr (nmol/L)	1235.62 ± 377.88	1402.76 ± 426.11	2.740	0.007
Non-HDL-C (mg/dL)	120.62 ± 34.74	131.50 ± 37.68	2.004	0.046
Non-HDL-C-corr (mg/dL)*	112.53 (84.79, 136.45)	116.96 (96.00, 147.17)	2.148	0.033

TG, triglyceride; ApoA1, apolipoprotein A1; ApoB, apolipoprotein B; TC-p, total particles of cholesterol; TC, total cholesterol; Lp(a), lipoprotein a; Lp(a)-p, particles of Lp(a); LDL-C, low-density lipoprotein cholesterol; LDL-p, particles of LDL; LDL-size, average diameter of LDL-p; LDL-p-corr and LDL-C-corr, corrected LDL-p and LDL-C; HDL-C, high-density lipoprotein cholesterol; HDL-p, particles of HDL; non-HDL-C, none high-density lipoprotein cholesterol; non-HDL-p, particles of non-HDL; non-HDL-p-corr and non-HDL-C-corr, corrected non-HDL-p and non-HDL-C

Lp(a) = Lp(a)-p*0.4167; LDL-p-corr = LDL-p – Lp(a)-p; LDL-C-corr = LDL-C – 0.3*Lp(a); non-HDL-p = TC-p – HDL-p; non-HDL-p-corr = non-HDL-p – Lp(a)-p; Non-HDL-C = TC – HDL-C; non-HDL-C-corr = non-HDL-C – 0.3*Lp(a)

TG, ApoB, TC-p, Lp(a)-p, Lp(a), LDL-C-size, HDL-C-p, HDL-C and non-HDL-C-corr were skew distribution and shown as median (25th percentile, 75th percentile). Before the Student’s t-test for the difference between groups, the nonnormal distribution variables were converted into natural logarithm form

^a^Overweight was defined as body mass index (BMI) > 28 (BMI = weight (Kg) / height (m))

^b^Family history was defined as the age of onset of coronary heart disease less than 55 for men and less than 65 for women, in the immediate family members of patients

^c^TIMI flow classification was used to evaluate coronary artery perfusion by coronary angiography. It was divided into grade 0 (no perfusion); Grade 1 (infiltration without perfusion); Grade 2 (partial perfusion) and Grade 3 (complete perfusion)

### Risk factors of CHD

**Fig. 2:**
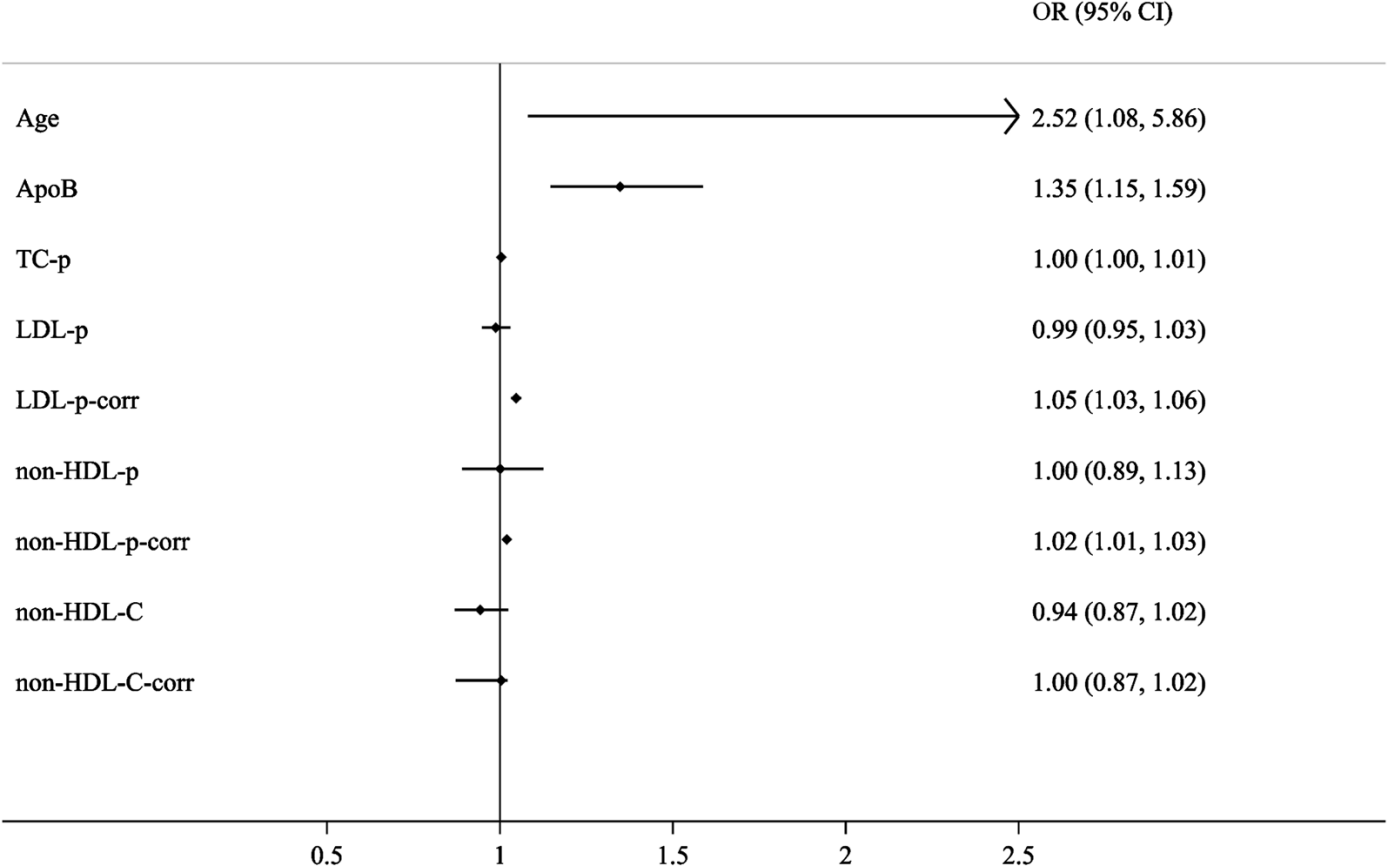
Forest plot of the multivariable logistic regression analysis. This forest plot was based on the multivariable logistic regression analysis of the risk factors of coronary heart disease in postmenopausal women. ApoB, apolipoprotein B; TC-p, total particles of cholesterol; LDL-p, particles of LDL; LDL-p-corr, corrected LDL-p; non-HDL-C, none high-density lipoprotein cholesterol; non-HDL-p, particles of non-HDL; non-HDL-p-corr and non-HDL-C-corr, corrected non-HDL-p and non-HDL-C. LDL-p-corr = LDL-p – particles of lipoprotein a [Lp(a)-p]; non-HDL-p = TC-p – HDL-p; non-HDL-p-corr = non-HDL-p – Lp(a)-p; Non-HDL-C = TC – HDL-C; non-HDL-C-corr = non-HDL-C – 0.3*Lp(a)

The multivariate analysis of risk factors associated with CHD was displayed in Fig. 2. Among the 9 variables, Age (hazard ratio (HR) 2.58, 95% confidence interval (CI) 1.08–5.86, *P* = 0.03), ApoB (HR 1.35, 95% CI 1.15–1.59, *P* < 0.001), LDL-p-corr (HR 1.05, 95% CI 1.03–1.06, *P* < 0.001) and non-HDL-p-corr (HR 1.02, 95% CI 1.01–1.03, *P* < 0.001) were risk factors of CHD.

### Correlation analysis of lipid indexes and Gensini score

**Fig. 3:**
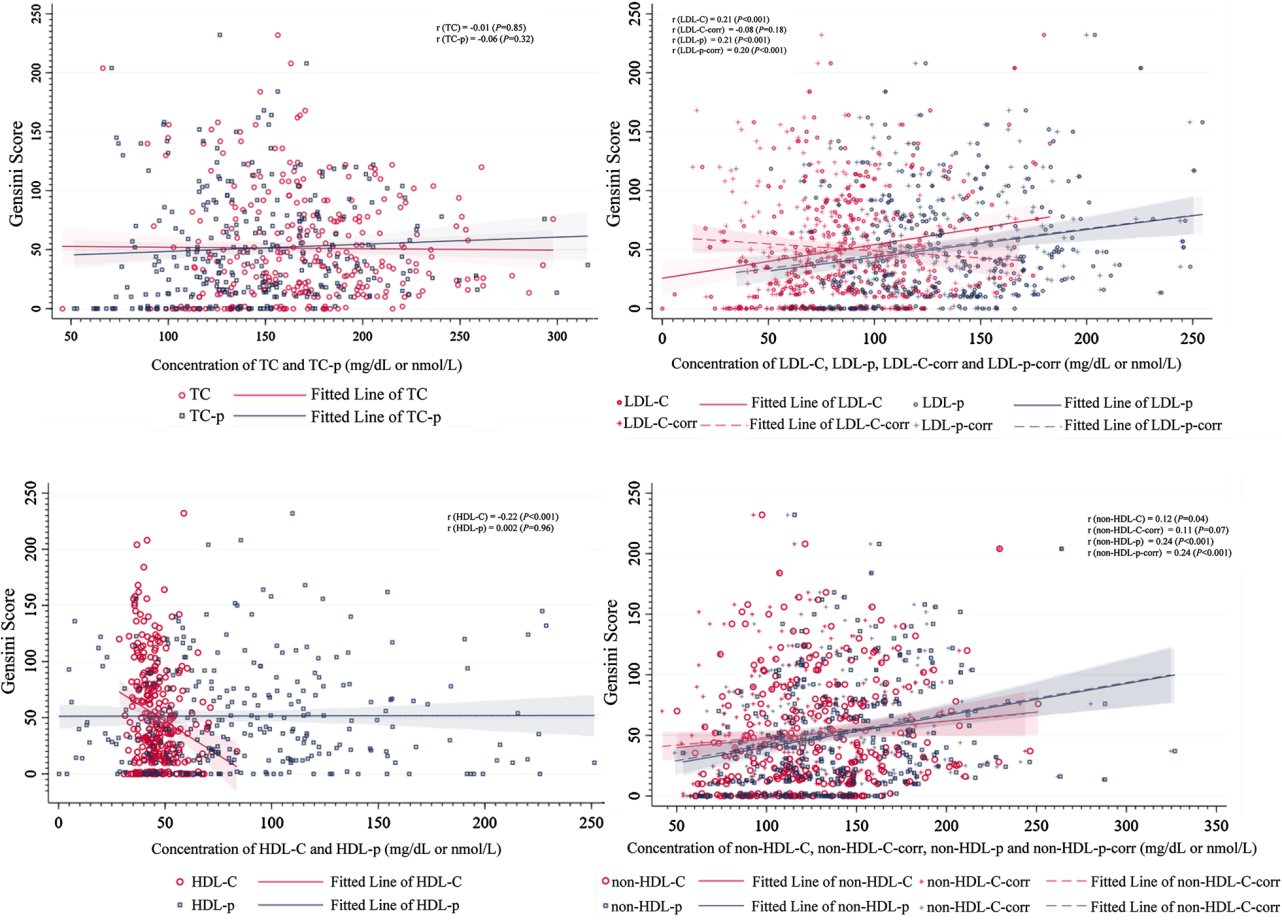
Spearman correlation analysis between routine, advanced lipid indexes and Genisi score. Associations between variables and Gensini were assessed with Spearman correlation analysis. TC, total cholesterol; TC-p, total particles of cholesterol; LDL-C, low-density lipoprotein cholesterol; LDL-p, particles of LDL; LDL-C-corr, the correction of LDL-C; LDL-p-corr, the correction of LDL-p; HDL-C, high-density lipoprotein cholesterol; HDL-p, particles of HDL; non-HDL-C-corr, correction of non-HDL-C; non-HDL-p, particles of non-HDL; non-HDL-p-corr, correction of non-HDL-p. LDL-C-corr = LDL-C – 0.3*lipoprotein a [Lp(a)]; LDL-p-corr = LDL-p – particles of Lp(a) [Lp(a)-p]; non-HDL-C = TC – HDL-C; non-HDL-C-corr = non-HDL-C – 0.3*Lp(a); non-HDL-p = TC-p – HDL-p; non-HDL-p-corr = non-HDL-p – Lp(a)-p

Figure 3 showed the Spearman correlation analysis of routine, advanced lipid indexes and Gensini score. In general, LDL-C, LDL-p, LDL-p-corr, HDL-C, non-HDL-C, non-HDL-p and non-HDL-p-corr were in linear correlation with Gensini score. The correlation coefficient r ranged in 0.203 of LDL-p-corr to 0.242 of non-HDL-C-p. There was no significant difference in the comparison between routine and advanced lipid items in the correlation analysis with Gensini.

### Predictive ability of routine and advanced lipid indexes for CHD

**Fig. 4:**
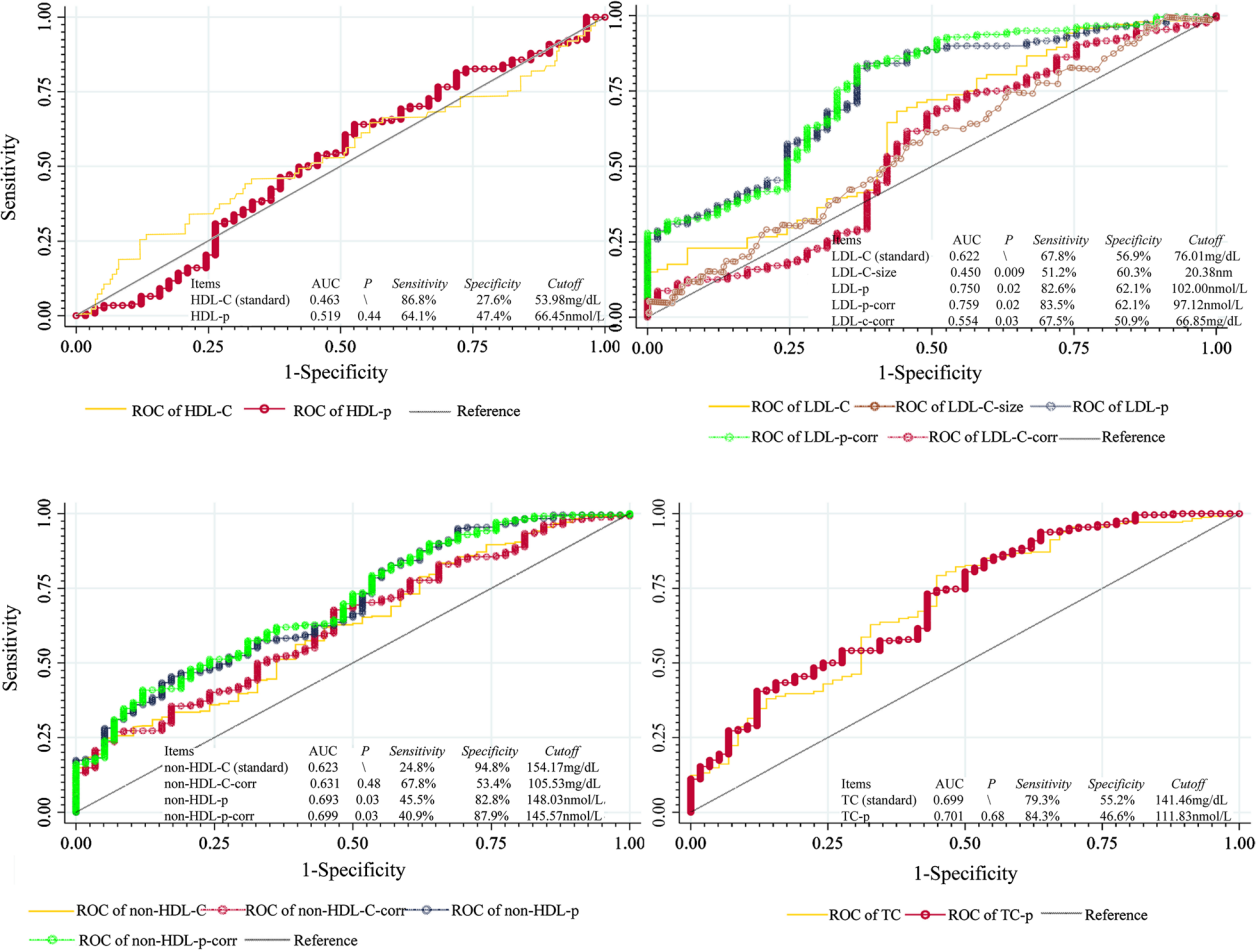
ROC plot of routine, advanced lipid indexes and Genisi score. HDL-C, high-density lipoprotein cholesterol; HDL-p, particles of HDL; LDL-C, low-density lipoprotein cholesterol; LDL-C-size, average diameter of LDL particles; LDL-p, particles of LDL; LDL-p-corr, the correction of LDL-p; LDL-C-corr, the correction of LDL-C; non-HDL-C-corr, correction of non-HDL-C; non-HDL-p, particles of non-HDL; non-HDL-p-corr, correction of non-HDL-p TC, total cholesterol; TC-p, total particles of cholesterol. LDL-C-corr = LDL-C – 0.3*lipoprotein a [Lp(a)]; LDL-p-corr = LDL-p – particles of Lp(a) [Lp(a)-p]; non-HDL-C = TC – HDL-C; non-HDL-C-corr = non-HDL-C – 0.3*Lp(a); non-HDL-p = TC-p – HDL-p; non-HDL-p-corr = non-HDL-p – Lp(a)-p

In the ROCs, displayed in Fig. 4, the most predictive lipid index for CHD was LDL-p-corr, with AUC = 0.759, cutoff of 97.12 nmol/L, while the weakest one was HDL-p with AUC = 0.519. The routine lipid items, HDL-C, LDL-C, non-HDL-C and TC were set as the standards in the comparison of the predictive powers. Advanced lipid indexes, LDL-p (*P* = 0.02) and LDL-p-corr (*P* = 0.02) were more predictive than the standard (LDL-C), while LDL-size (*P* = 0.009) and LDL-C-corr (*P* = 0.03) were less than LDL-C. The predictive capability of non-HDL-p (*P* = 0.03) and non-HDL-p-corr (*P* = 0.03) were also stronger than standard (non-HDL-C). Among all lipid indexes, the most sensitive one for CHD was HDL-C (sensitivity, 86.8%), and the most specific was non-HDL-C (specificity, 94.8%).

## Discussion

The recently published study showed a difference of disability adjusted life year (DALY) due to CVD between sexes that the total CVD DALYs of male were significantly higher than female at the age of 30–60, but the relation reversed after 80–84 [20]. In this study, we found age as the most important risk factor of CHD in a group of elderly, postmenopausal women. It is known to all that the morbidity of CHD, hypertension, DM and metabolic syndrome increased with age. Besides, age is also a factor influencing the efficacy of hormone replacement therapy for postmenopausal women [21]. Recently published 10-year follow-up results of the BRECARD Study [22] indicated that the higher the content of breast adipose tissue, the higher the incidence of MACE in pre-menopausal women. In addition to the traditional risk factors, adipokines may play an important role in atherosclerosis process in elder people [23]. We hypothesize that adipokines are involved in lipid metabolism, fat deposition (increased body mass index) and redistribution (at accumulation in breast, abdomen and other trunk positions) and inflammation, which contribute to the abnormal lipid metabolism and atherosclerosis.

The results of this study also indicated that compared with routine lipid indexes LDL-C and non-HDL-C, the advanced ones, LDL-p, LDL-p-corr, non-HDL-p and non-HDL-p-corr were more predictive for CHD in postmenopausal women. Due to the similarity of Lp(a) and LDL-C, both the traditional lipid panel and NMR reported LDL-C was a mixture of LDL-C and Lp(a). It was reported that 30%-45% LDL-C in traditional lipid panel was contributed by Lp(a) and in more extreme cases, the majority of LDL-C was carried by Lp(a) when LDL-C less than 25 mg/dL [24]. To distinguish the pathogenic role of Lp(a) and LDL-C in atherosclerosis, 0.3*Lp(a) (mg/dL) and LDL-C-corr (LDL-C – 0.3*Lp(a)) was used in several studies, as cholesterol account for 30%-45% of Lp(a) particle [18, 25]. Those two lipid items are focused on the pathogenic effect of cholesterol in both Lp(a) and LDL-C, but the LDL-p and Lp(a)-p are also strong predictors of CHD. In this study, we measured Lp(a)-p in nmol/L with Tina-quant Lipoprotein (a) Gen.2, a latex coated antibody of apo(a), and the accuracy were higher among six common commercial assays [26]. Furthermore, we made an innovation of LDL-p-corr and non-HDL-p-corr, which mean pure particles of LDL and non-HDL. As we know, these two lipid items initiated by us have not been studied before. Our study proved that LDL-p-corr and non-HDL-p-corr were risk factors of CHD and significantly correlated with Gensini score in postmenopausal women.

In the ROCs, we found HDL-C was the traditional blood lipid item with the weakest predictive ability for CHD, with the AUC under 0.50. It was consistent with the current view of the relationship between HDL and CHD [27]. In the comparison of predictive abilities between traditional and advanced lipid items, the predictive abilities of LDL-p, LDL-p-corr, non-HDL-p and non-HDL-p-corr were significantly stronger than the traditional lipid items, LDL-C and non-HDL-C. That indicated the particles of LDL and non-HDL may be better lipid indexes in CHD prevention. However, whether this finding is a general feature of the population or only confined to postmenopausal women remains to be confirmed by further studies.

There are several limitations of this study. First, over 80% of the study population were diagnosed with CHD, the result can’t represent the general characteristics of postmenopausal women. Second, menopause status came from the patients’ reports, more than 30% of the study population could not provide accurate menopause time, we took age as the risk factor of CHD, and it will be more precise if the postmenopausal time or serum sex hormones were available. But the age was not well matched between two groups and the differences of CHD risk factors and metabolic indexes can’t be ruled out whether it is caused by age. Third, limited by the cross-sectional design, reversal causality is possible. For example, we couldn’t confirm whether ApoB, LDL-p-corr and non-HDL-p-corr were pathogenic factors of CHD or just the metabolic characteristics of postmenopausal women with CHD. Finally, further research is needed to confirm the stability of lipid indexes in frozen samples and repeatability of the advanced lipid measurement.

## Conclusion

In postmenopausal females, age, ApoB, corrected particles of LDL and non-HDL are risk factors of CHD. LDL-C, non-HDL-C, LDL-p-corr and non-HDL-p-corr were positively correlated with coronary plaque burden, while HDL-C was negatively correlated. Compared with LDL-C and non-HDL-C, the particles and the corrected particles of LDL and non-HDL were better predictors for CHD.

## Data Availability

All data generated or analyzed during this study are included in this published article.

## Abbreviations

CVD: Cardiovascular disease
ASCVD: Atherosclerotic cardiovascular disease
CAD: Coronary atherosclerotic disease
CHD: Coronary heart disease
DM: Diabetes mellitus
MS: Metabolic syndrome
TG: Triglycerides
TC: Total cholesterol
TC-p: Particles of cholesterol
VLDL: Very-low-density lipoprotein
IDL: Intermediate-density lipoprotein
LDL-C: Low-density lipoprotein cholesterol
LDL-p: Particles of LDL
LDL-size: Average diameter of LDL
LDL-C-corr: Corrected LDL-C
LDL-p-corr: Corrected particles of LDL
HDL-C: High-density lipoprotein cholesterol
HDL-p: Particles of HDL
non-HDL-C: Non-high-density lipoprotein cholesterol
non-HDL-p: Particles of non-HDL
non-HDL-C-corr: Corrected non-HDL-C
non-HDL-p-corr: Corrected particles of non-HDL
Lp(a): Lipoprotein a
Lp(a)-p: Particles of Lp(a)
ApoB: Apolipoprotein B
E2: Estradiol
PCSK9: Proprotein convertase subtilisin/kexin type-9
C-RP: C-reactive protein
NMR: Nuclear magnetic resonance
CAG: Coronary angiography
PCI: Percutaneous coronary intervention
CABG: Coronary artery bypass graft
LVEF: Left ventricular ejection fraction
